# Cardiac metastasis of anaplastic meningioma: a diagnostic challenge with multimodality imaging findings

**DOI:** 10.1093/ehjcr/ytad551

**Published:** 2023-11-07

**Authors:** José M Sánchez-Moreno, Jesús López-Munoz, Elvira Ruiz-Castellano, Juan Emilio Alcalá-López

**Affiliations:** Cardiology Department, Hospital Universitario Virgen de las Nieves, Avda. Fuerzas Armadas 2, Granada 18014, Spain; Cardiology Department, Hospital Universitario Virgen de las Nieves, Avda. Fuerzas Armadas 2, Granada 18014, Spain; Radiology Department, Hospital Universitario Virgen de las Nieves, Granada, Spain; Cardiology Department, Hospital Universitario Virgen de las Nieves, Avda. Fuerzas Armadas 2, Granada 18014, Spain

A 51-year-old woman with a history of smoking, Factor XII deficiency, and left temporal lobe anaplastic meningioma operated 1 month before was admitted due to dyspnoea.

Acute pulmonary embolism was ruled out by chest cardiac computed tomography angiography (CTA), but a high-density, hypocaptant, and heterogeneous mass suggestive of either a thrombus or a tumour lesion in the left atrium (LA) was described (*Figure 1A*).

**Figure 1:**
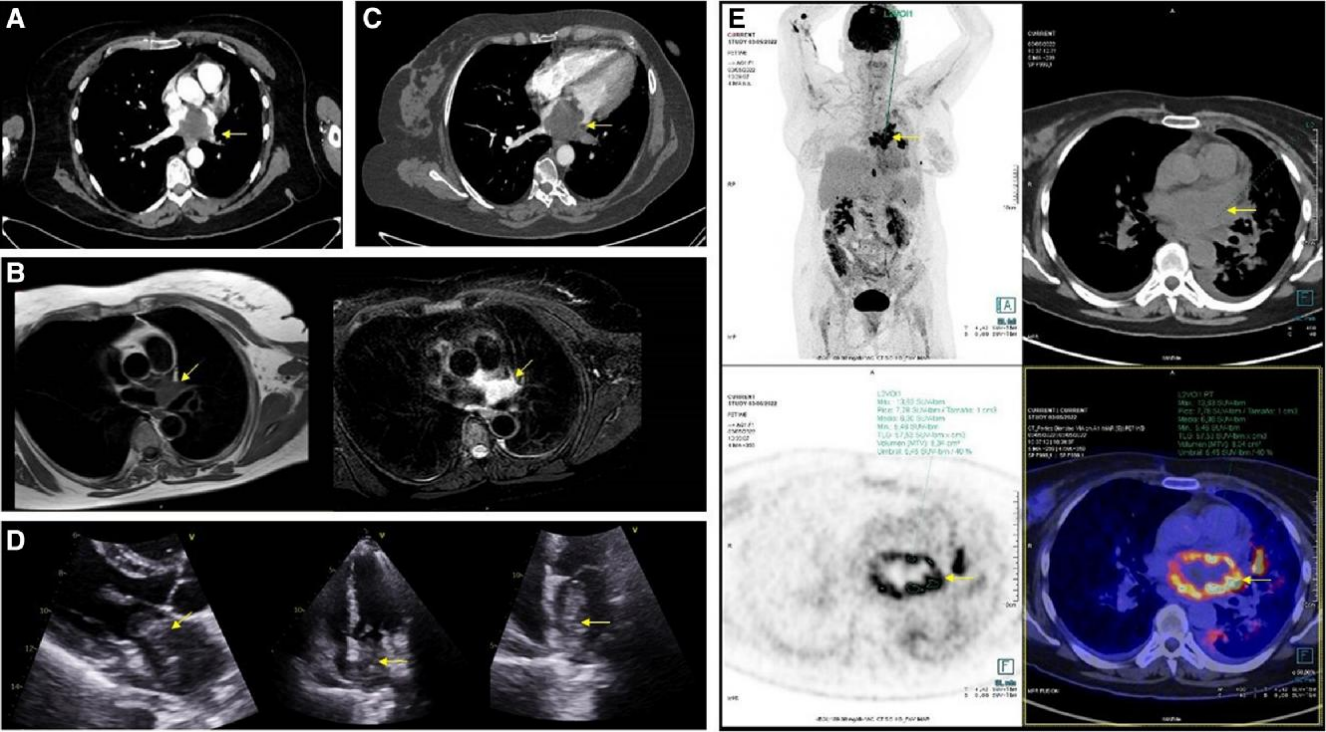
(*A*) Initial computed tomography angiography showing a mass within the left atrium suggestive of either a thrombus or a tumour lesion. (*B*) Cardiovascular magnetic resonance showing a heterogeneous image, isointense on T1-weighted images and hyperintense on T2-weighted image, with no late gadolinium enhancement, which protruded into the left atrium from the outlet of the left upper pulmonary vein. (*C*) Second computed tomography angiography showing a growth of the mass size, which occupies almost the entire left atrium. (*D*) Transthoracic echocardiogram showing a 55 × 45 mm non-pedunculated multilobulated motionless mass of inhomogeneous echogenicity attached to the atrial posterior wall extending into the PVs and the mitral valve. (*E*) Body FDG PET/computed tomography angiography showing images compatible with metastatic implants in lungs, bones, and muscles and left atrium mass uptake. PV, pulmonary vein; FDG, fluorodeoxyglucose; PET, positron emission tomography.

Cardiovascular magnetic resonance (CMR) was carried out showing a heterogeneous image, isointense on T1-weighted images (WIs) and hyperintense on T2-WI, with no late gadolinium enhancement, which protruded into the LA from the outlet of the left upper pulmonary vein (*Figure 1B*).

These findings were misinterpreted as compatible with thrombus, and the patient was discharged with 14 000 UI/24 h subcutaneous tinzaparin as anticoagulation therapy.

Three months later, a meningioma recurrence in the left frontal lobe was treated surgically with a periprocedural temporary suspension of tinzaparin. A week after discharge, the patient was readmitted because of rapidly progressive dyspnoea. A new chest CTA allowed to rule out pulmonary embolism, but the size of the mass had increased, occupying almost the entire LA (*Figure 1C*).

Transthoracic echocardiogram (TTE) showed a 55 × 45 mm non-pedunculated multilobulated motionless mass of inhomogeneous echogenicity attached to the atrial posterior wall extending into the pulmonary veins and the mitral valve without impairment on mitral inflow (*Figure 1D*).

Body fluorodeoxyglucose-positron emission tomography-computed tomography displayed images compatible with metastatic implants in lungs, bones, and muscles (*Figure 1E*). The LA mass uptake was similar to a biopsied paravertebral lesion in which histological examination proved anaplastic meningioma metastasis. Furthermore, the rapid growth and characteristics of the mass by multimodal imaging support the diagnosis of cardiac metastasis of meningioma. The patient was referred to medical oncology and is currently undergoing chemotherapy.

Despite the fact that thrombi are the most common cardiac masses, the second most common aetiology is neoplastic, with a secondary/primary tumour ratio of 20:1. Approximately 10% of primary cardiac tumours are malignant and 90% benign.^1^ The common site for metastasis is the right heart but is very unlikely to metastasize to the LA, although a similar case of metastatic meningioma extending to the LA through the pulmonary veins has been previously described.^2^ This report emphasizes the rarity of this condition and the importance of a multimodality imaging approach in the assessment of intracardiac masses combining the different information from TTE, CTA, CMR, and nuclear imaging.^3^

## Data Availability

No new data were generated or analysed in support of this research.

## Notes

**Consent:** Patient consent obtained in accordance with COPE guidelines.

**Funding:** None declared.
